# Supplementary material for: Alternative splicing of coq-2 controls the levels of rhodoquinone in animals
Source: eLife. 2020 Aug 3;9:e56376. doi: 10.7554/eLife.56376 (PMC7434440; doi:10.7554/eLife.56376)
Supplement: Supplementary file 1. [file elife-56376-supp1.elegansstrains.docx]

**SUPPLEMENTARY FILE 1:** *C. elegans* strains

**A. Strain PHX1715 (*coq-2(syb1715)*), deletion mutant in *coq-2* exon 6A**

1) *coq-2* wild-type sequence of exon 6A (capital letters) and 30bp flanking sequence (red):

atctcttacactctctctatcatagtacagGACTCACGTTCAATTGGGGCGCTCTTCTTGGATGGTGTGCGCTGAAAGGTGATTTGTCGTCTAGTGCACCGTTTTGGATGTATGCAGCTGCACTTCAATGGACACTGATCTACGACACTATCTATGCACATCAGgtggcctatttggggattgggactcttttg

2) Deleted sequence (-134 bp):

GACTCACGTTCAATTGGGGCGCTCTTCTTGGATGGTGTGCGCTGAAAGGTGATTTGTCGTCTAGTGCACCGTTTTGGATGTATGCAGCTGCACTTCAATGGACACTGATCTACGACACTATCTATGCACATCAG

3) sgRNA target sites used for strains PHX1715 (PAM motives underlined):

sg1：acagGACTCACGTTCAATTGGGG
sg2：TGTATGCAGCTGCACTTCAATGG
sg3：GCACATCAGgtggcctatttggg

4) PCR and sequencing primers:

GUC01-ko-f: AAATAAGGTTTTCCCGCCAG

GUC01-ko-r: GTCGACTTGACACCGATCAT

**B. Strain PHX1721 (*coq-2(syb1721)*), deletion mutant in *coq-2* exon 6E**

1) *coq-2* wild-type sequence of exon 6E (capital letters) and 30bp flanking sequence (red):

aacctagtctgtcgttttgtacgattgcagGTGCCACCCTAAACTGGAGTGTGCTGATAGCGTGGGCAGAATTGGGCCATTTCAATGATTTTGGCATCTTTTTGCCACTCTACACTGCCACCATCCTGCACACGGTCATCTACGACACTATTTATAGTCATCAGgtagagctaagaaaagcaggaaaaaagtat

2) Deleted sequence (-134 bp):

GTGCCACCCTAAACTGGAGTGTGCTGATAGCGTGGGCAGAATTGGGCCATTTCAATGATTTTGGCATCTTTTTGCCACTCTACACTGCCACCATCCTGCACACGGTCATCTACGACACTATTTATAGTCATCAG

3) sgRNA target sites used for strain PHX1721 (PAM motives underlined):

sg1：CCACCCTAAACTGGAGTGTGCTG

sg2：CCTGCACACGGTCATCTACGACA

4) PCR and sequencing primers:

GUC02-ko-f: CAAAAACAAGTACGGTTCCC

GUC02-ko-r: CGCAGCACGGTATGAGACTTT
