# Supplementary material for: Alternative splicing of coq-2 controls the levels of rhodoquinone in animals
Source: eLife. 2020 Aug 3;9:e56376. doi: 10.7554/eLife.56376 (PMC7434440; doi:10.7554/eLife.56376)
Supplement: Supplementary file 2. [file elife-56376-supp2.docx]

**SUPPLEMENTARY FILE 2**. Statistical analysis of RQ_9_ and UQ_9_ levels in *coq-2* mutant strains

| Strain | Avg pmol RQ_9_/mg pellet | p value (N = 4) | | Avg pmol UQ_9_/mg pellet | p value (N = 4) |
| --- | --- | --- | --- | --- | --- |
| N2 | 3.30 ± 0.74 | |  | 16.55 ± 4.76 |  |
| *coq-2*∆*6a* | 5.43 ± 1.35 | | 0.016 | 2.81 ± 0.27 | < 0.001 |
| *coq-2*∆*6e* | 0.40 ± 0.08 | | < 0.001 | 20.23 ± 3.54 | 0.130 |
